# Construction and validation of a novel prognostic model for intrahepatic cholangiocarcinoma based on a combined scoring system of systemic immune-inflammation index and albumin-bilirubin: a multicenter study

**DOI:** 10.3389/fonc.2023.1239375

**Published:** 2023-09-28

**Authors:** Haofeng Zhang, Qingshan Li, Guan Huang, Zhenwei Yang, Kunlun Chen, Bo Meng, Haibo Yu

**Affiliations:** ^1^ Department of Hepatobiliary and Pancreatic Surgery, People’s Hospital of Zhengzhou University, Zhengzhou, China; ^2^ Department of Hepatobiliary and Pancreatic Surgery, Henan Provincial People’s Hospital, Zhengzhou, China; ^3^ Department of Hepatobiliary and Pancreatic Surgery, People’s Hospital of Henan University, Zhengzhou, China; ^4^ Department of Hepatobiliary and Pancreatic Surgery, The First Affiliated Hospital of Zhengzhou University, Zhengzhou, China; ^5^ Department of Hepatobiliary and Pancreatic Surgery, Cancer Hospital of Zhengzhou University, Zhengzhou, China

**Keywords:** intrahepatic cholangiocarcinoma, SII, ALBI, SII+ALBI grade, nomogram, prognosis

## Abstract

**Background:**

The degree of inflammation and immune status is widely recognized to be associated with intrahepatic cholangiocarcinoma (ICC) and is closely linked to poor postoperative survival. The purpose of this study was to evaluate whether the systemic immune-inflammatory index (SII) and the albumin bilirubin (ALBI) grade together exhibit better predictive strength compared to SII and ALBI separately in patients with ICC undergoing curative surgical resection.

**Methods:**

A retrospective analysis was performed on a cohort of 374 patients with histologically confirmed ICC who underwent curative surgical resection from January 2016 to January 2020 at three medical centers. The cohort was divided into a training set comprising 258 patients and a validation set consisting of 116 patients. Subsequently, the prognostic predictive abilities of three indicators, namely SII, ALBI, and SII+ALBI grade, were evaluated. Independent risk factors were identified through univariate and multivariate analyses. The identified independent risk factors were then utilized to construct a nomogram prediction model, and the predictive strength of the nomogram prediction model was assessed through Receiver Operating Characteristic (ROC) survival curves and calibration curves.

**Results:**

Univariate analysis of the training set, consisting of 258 eligible patients with ICC, revealed that SII, ALBI, and SII+ALBI grade were significant prognostic factors for overall survival (OS) and recurrence-free survival (RFS) (p < 0.05). Multivariate analysis revealed the independent significance of SII+ALBI grade as a risk factor for postoperative OS and RFS (p < 0.05). Furthermore, we conducted an analysis of the correlation between SII, ALBI, SII+ALBI grade, and clinical features, indicating that SII+ALBI grade exhibited stronger associations with clinical and pathological characteristics compared to SII and ALBI. We constructed a predictive model for postoperative survival in ICC based on SII+ALBI grade, as determined by the results of multivariate analysis. Evaluation of the model’s predictive strength was performed through ROC survival curves and calibration curves in the training set and validation set, revealing favorable predictive performance.

**Conclusion:**

The SII+ALBI grade, a novel classification based on inflammatory and immune status, serves as a reliable prognostic indicator for postoperative OS and RFS in patients with ICC.

## 1 Introduction

Intrahepatic cholangiocarcinoma (ICC) is the second most prevalent primary liver cancer distinguished by its aggressive nature, accounting for approximately 15–20% of all biliary malignancies (1, 2). The worldwide incidence of ICC has been consistently rising at a yearly rate of 15% over the past few decades (1). Curative surgical resection currently stands as the gold-standard treatment for ICC. However, only about 20%–40% of patients who get curative surgical resection survive 5 years or more (3, 4). Therefore, the identification of novel prognostic indicators for distinguishing ICC patients who would benefit from curative surgical resection is crucial for developing personalized treatment strategies.

Increasing evidence suggests that in addition to common factors such as lymph node metastasis, tumor size, and vascular invasion, nutritional status and inflammatory levels play a significant predictive role in the prognosis of curative surgical resection for tumors (5, 6). Among them, the Systemic Immune-Inflammation Index (SII) is a fresh quantitative indicator used to assess individual immune status and inflammation levels (7, 8). It is calculated based on parameters such as platelet, neutrophil, and lymphocyte counts. SII is frequently used to assess patients’ preoperative nutritional status and precisely evaluate their individual surgical risks (8). Additionally, the Albumin–bilirubin (ALBI) grade is a composite indicator that comprehensively evaluates patients’ liver function and reserves. Its introduction was first compared to Child-Pugh classification in hepatocellular carcinoma (HCC) patients in 2015, demonstrating superior predictive capability for survival following liver resection and postoperative liver failure (9). A growing body of literature indicates a close association between SII, ALBI, and the prediction of prognosis and survival in patients with HCC, ICC, and other malignancies (9–13). However, whether the combined application of SII and ALBI can improve the prognostic prediction in patients with ICC remains inconclusive. This research seeks to identify the combined application of SII and ALBI in predicting postoperative survival after curative resection for ICC and attempt to construct a survival prognostic model based on SII and ALBI.

## 2 Materials and methods

### 2.1 Patient selection

This study included all patients who received curative surgical resection for ICC between January 2016 and January 2020 at People’s Hospital of Zhengzhou University, Cancer Hospital of Zhengzhou University, and The First Affiliated Hospital of Zhengzhou University. Following were the inclusion criteria: 1) Patients whose pathological confirmation with ICC followed a curative surgical resection; 2) Patients aged 18 years or older; 3) No prior anticancer treatment before surgery; 4) No concurrent occurrence of other malignant tumors. Following were the exclusion criteria: 1) Perioperative mortality; 2) Patients with hematological disorders and autoimmune diseases; 3) Incomplete clinical or laboratory data; 4) Patients requiring a second surgery for tumor recurrence; 5) Incomplete follow-up information. 258 patients from People’s Hospital of Zhengzhou University and Cancer Hospital of Zhengzhou University were chosen as the training set, while a total of 116 patients from The First Affiliated Hospital of Zhengzhou University were chosen as the validation set. The 8th edition of the American Joint Committee on Cancer (AJCC) staging method was used to evaluate all patients who were included, and all patients were monitored until January 2023.

This study received ethical approval from the Institutional Review Boards of Zhengzhou University People’s Hospital (Ref No. 2023-012), Zhengzhou University Cancer Hospital (Ref No. 2023-203), and Zhengzhou University First Affiliated Hospital (2021-KY-1137-002). Written informed consent was obtained from all patients prior to their participation in the study.

### 2.2 Clinical variables

Patient clinical and pathological data included age, gender, HBV infection, obstructive jaundice, tumor differentiation, tumor number, tumor size, perineural invasion, microvascular invasion, and the AJCC 8^th^ TNM Stage. Laboratory test results were collected from one week before surgery, including carbohydrate antigen 19-9 (CA19-9), carcinoembryonic antigen (CEA), alpha-fetoprotein (AFP), alanine transaminase (ALT), aspartate transaminase (AST), albumin, bilirubin, white blood cell count (WBC), lymphocyte count (LY), neutrophil count (NEUT), platelet count (PLT), hemoglobin (HGB), prothrombin time (PT), international normalized ratio (INR) and activated partial thromboplastin time (APTT). Additionally, the calculation methods for the two immune-inflammatory markers, ALBI and SII, were as follows: ALBI = log_10_bilirubin (mol/L) * 0.66 - albumin (g/L) * 0.085, SII = platelet count * neutrophil count/lymphocyte count. Subsequently, Subsequently, the X-tile software (Yale University, New Haven, CT, USA) was employed to compute the optimal cutoff values for overall survival (OS) and recurrence-free survival (RFS) with respect to SII and ALBI. Based on the results, ALBI ≥ -2.50 was defined as the high ALBI group, and ALBI < -2.50 as the low ALBI group. Similarly, SII ≥ 470 was defined as the high SII group, and SII < 470 as the low SII group. In the subsequent analysis, the combination of low SII and low ALBI was defined as SII+ALBI Grade A, the combination of high SII and high ALBI was defined as SII+ALBI Grade C, and the remaining combinations were defined as SII+ALBI Grade B.

### 2.3 Statistical analysis

The Kolmogorov-Smirnov test was used in research to determine if continuous variables were normally distributed. Mean and standard deviation (SD) were used to represent normally distributed data, whereas interquartile range (IQR) was used to represent non-normally distributed variables. For group comparisons, the Mann-Whitney rank sum test and the student t-test were used. The baseline features of categorical variables were compared using the chi-square test and Fisher’s exact test. Cox proportional hazards regression analysis was used for the univariate analysis. Cox backward stepwise regression models were employed for the multivariate analysis. GraphPad Prism (version 8.0) was used to create Kaplan-Meier survival curves for OS and RFS based on the grouping of ALBI, SII, and ALBI+SII. Additionally, ROC survival curves were drawn, and the three groups’ areas under the curve (AUC) were contrasted. Statistical significance was defined as p<0.05.

### 2.4 Follow-up

For the included patients, follow-up began after the surgical procedure. Within the first year postoperatively, monthly follow-up visits were conducted, followed by follow-up visits every three months for the next two years. The last follow-up was performed on January 2023. Overall survival was determined as the interval between the date of curative surgical resection and the last examination or the date of death from any cause. Recurrence-free survival was determined as the interval between the date of curative surgical resection and the most recent follow-up, the occurrence of tumor recurrence or advancement in any way, or the patient’s death for any reason.

### 2.5 Development and assessment of nomogram

Based on the results of the Cox backward stepwise regression model, predictive models for OS and RFS were constructed using nomogram models. The accuracy of the models was assessed by plotting ROC survival curves and calibration curves for the training and validation sets based on the models. The construction and evaluation of the models were performed using R software (version 4.2.1).

## 3 Result

A total of 374 patients (172 male and 202 female) who underwent curative surgical resection for pathologically confirmed ICC from January 2016 to January 2020 were included in this study. The median age of the patients was 59 years, ranging from 28 to 80 years. The median follow-up time was 12 months (1-91 months). The 1-year, 2-year, and 3-year OS rates were 52.1%, 23.3%, and 10.9%, respectively. The 1-year, 2-year, and 3-year RFS rates were 29.2%, 15.5%, and 5.3%, respectively. As can be seen in **Table 1**, the baseline data and clinicopathological traits of the training set (n=258) and validation set (n=116) were examined for their association. The two cohorts’ distributions were balanced (p>0.05).

**Table 1:** Comparison of clinicopathological characteristics in training and validation sets.

Variables	All patients (n=374)	Training set (n=258)	Validation set (n=116)	p value
Sex				0.884
Male	172(46.0%)	118(45.7%)	54(46.6%)	
Female	202(54.0%)	140(54.3%)	62(53.4%)	
Age (years)				0.076
≤65	250(66.8%)	165(64.0%)	85(73.3%)	
>65	124(33.2%)	93(36.0%)	31(26.7%)	
Obstructive jaundice				0.965
No	310(82.9%)	214(82.9%)	96(82.8%)	
Yes	64(17.1%))	44(17.1%)	20(17.2%)	
HBV infection				0.467
No	239(63.9%)	168(65.1%)	71(61.2%)	
Yes	135(36.1%)	90(34.9%)	45(38.8%)	
AFP (ng/ml)				0.085
<20	309(82.6%)	219(84.9%)	90(77.6%)	
≥20	65(17.4%)	39(15.1%)	26(22.4%)	
CEA (ng/ml)				0.491
<5	242(64.7%)	164(63.6%)	78(67.2%)	
≥5	132(35.3%)	94(36.4%)	38(32.8%)	
CA19-9 (U/ml)				0.387
<37	149(39.8%)	99(38.4%)	50(43.1%)	
≥37	225(60.2%)	159(61.6%)	66(56.9%)	
Child–Pugh Grade				0.639
Grade A	334(89.3%)	233(90.3%)	101(87.1%)	
Grade B	40(10.7%)	25(9.7%)	15(12.9%)	
Tumor number				0.995
= 1	303(81.0%)	209(81.0%)	94(81.0%)	
>1	71(19.0%)	49(19.0%)	22(19.0%)	
Tumor size				0.366
<5.0cm	158(42.2%)	105(40.7%)	53(45.7%)	
≥5.0cm	216(57.8%)	153(59.3%)	63(54.3%)	
Tumor differentiation				0.627
Well	36(9.6%)	27(10.5%)	9(7.8%)	
Moderate	280(74.9%)	193(74.8%)	87(75.0%)	
Poor	58(15.4%)	38(14.7%)	20(17.2%)	
Perineural invasion				0.109
No	194(51.9%)	141(54.7%)	53(45.7%)	
Yes	180(48.1%)	117(45.3%)	63(54.3%)	
Microvascular invasion				0.727
No	205(54.8%)	143(55.4%)	62(53.4%)	
Yes	169(45.2%)	115(44.6%)	54(46.6%)	
AJCC 8th edition T stage				0.053
T_1a_/T_1b_	171(45.7%)	117(45.3%)	54(46.6%)	
T_2_	147(39.3%)	95(36.8%)	52(44.8%)	
T_3_/T_4_	56(15.0%)	46(17.8%)	10(8.6%)	
AJCC 8th edition N stage				0.252
N_0_	279(74.6%)	188(72.9%)	91(78.4%)	
N_1_	95(25.4%)	70(27.1%)	25(21.6%)	
AJCC 8th edition M stage				0.311
M_0_	368(98.4%)	255(98.8%)	113(97.4%)	
M_1_	6(1.6%)	3(1.2%)	3(2.6%)	
ALT (ng/ml)	53(45-61)	50(44-59)	60(44-75)	0.259
AST (ng/ml)	48(42-53)	45(38-52)	53(41-65)	0.222
Albumin (ng/ml)	40.54(39.90-41.18)	40.80(40.05-41.54)	39.98(38.75-41.20)	0.245
Bilirubin (ng/ml)	28.16(22.35-33.97)	26.88(20.08-33.68)	31.00(19.79-42.20)	0.520
PT (s)	12.30(12.17-12.44)	12.27(12.11-12.44)	12.37(12.13-12.62)	0.509
INR	1.21(1.00-1.41)	1.17(0.95-1.39)	1.28(0.83-1.74)	0.611
APTT (s)	32.00(31.36-32.65)	31.92(31.10-32.74)	32.18(31.16-33.20)	0.718
WBC (10^9^/L)	6.88(6.59-7.16)	6.76(6.44-7.09)	7.13(6.55-7.70)	0.248
HGB (g/L)	131(129-133)	130(128-133)	131(128-135)	0.710
NEUT (10^9^/L)	5.19(4.65-5.72)	4.94(4.38-5.51)	5.73(4.54-6.92)	0.183
LY (10^9^/L)	1.66(1.43-1.88)	1.71(1.39-2.03)	1.53(1.41-1.65)	0.470
PLT (10^9^/L)	215(207-223)	215(205-225)	215(200-231)	0.946
SII	706(741-946)	796(685-907)	950(728-1172)	0.172
ALBI	-2.67(-2.74 - -2.60)	-2.70(-2.77 – -2.62)	-2.60(-2.73 – -2.48)	0.207

### 3.1 Survival analysis for OS and RFS

**Figure 1:**
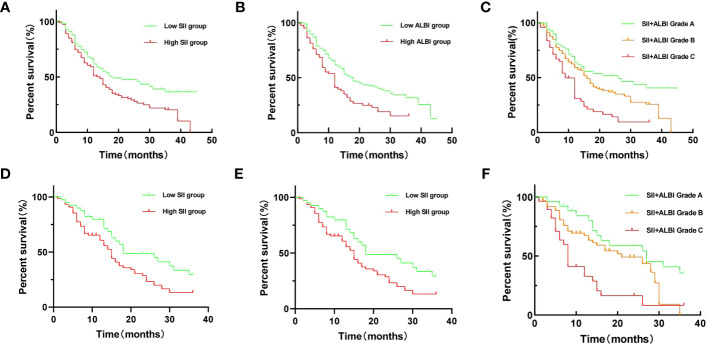
Kaplan–Meier overall survival (OS) curves of patients with intrahepatic cholangiocarcinoma (ICC) after radical resection according to different prognostic factors in the training set and validation set. **(A–C)** Kaplan–Meier OS curves according to the Systemic Immune-Inflammation Index (SII)**(A)**, albumin–bilirubin (ALBI) **(B)**, and SII+ALBI grade **(C)** of training set. **(D–F)** Kaplan–Meier OS curves according to SII **(A)**, ALBI **(B)**, and SII+ALBI grade **(C)** of validation set.

**Figure 2:**
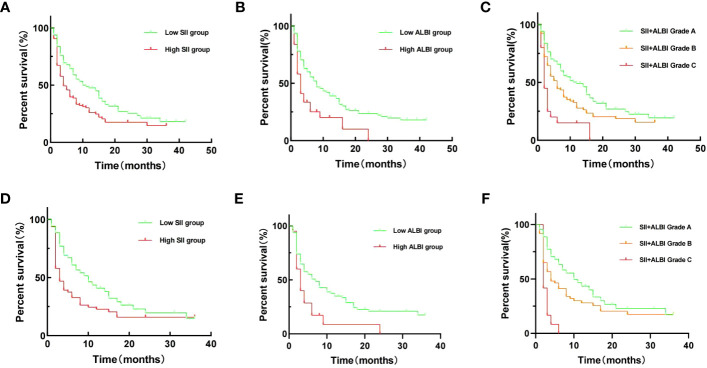
Kaplan–Meier recurrence-free survival (RFS) curves of patients with intrahepatic cholangiocarcinoma (ICC) after radical resection according to different prognostic factors in the training set and validation set. **(A–C)** Kaplan–Meier OS curves according to the Systemic Immune-Inflammation Index (SII) **(A)**, albumin–bilirubin (ALBI) **(B)**, and SII+ALBI grade **(C)** of training set. **(D–F)** Kaplan–Meier OS curves according to SII **(A)**, ALBI **(B)**, and SII+ALBI grade **(C)** of validation set.

Through univariate survival analysis of the included variables, we found that SII [OS: hazard ratio (HR) = 1.574, 95% CI = 1.126-2.201, p = 0.008; RFS: HR = 1.590, 95% CI = 1.181-2.140, p = 0.002], ALBI [OS: HR = 1.692, 95% CI = 1.220-2.346, p = 0.002; RFS: HR = 1.980, 95% CI = 1.291-3.038, p = 0.002], and SII+ALBI grade [OS: HR = 2.717, 95% CI = 1.701-4.341, p < 0.001; RFS: HR = 3.078, 95% CI = 1.822-5.198, p < 0.001] were prognostic factors for OS and RFS in patients with ICC after surgical resection (**Figures 1**, **2**). Additionally, the results of the multivariate survival analysis also indicated that SII+ALBI grade [OS: HR = 2.230, 95% CI = 1.371-3.628, p = 0.001; RFS: HR = 2.355, 95% CI = 1.359-4.082, p = 0.001] was an independent risk factor for OS and RFS in postoperative ICC patients (**Figures 1**, **2**). The detailed results of the univariate and multivariate analyses are presented in **Table 2**.

**Table 2:** Univariate and multivariate analyses of the prognosis for intrahepatic cholangiocarcinoma (ICC) after radical resection in the training set.

Variables	OS				RFS			
	Univariate analysis		Multivariate analysis		Univariate analysis		Multivariate analysis	
	HR (95%CI)	p value	HR (95%CI)	p value	HR (95%CI)	p value	HR (95%CI)	p value
Sex Female vs. Male	0.918(0.672-1.256)	0.594			1.040(0.773-1.400)	0.794		
Age (years) >65 vs. ≤65	0.947(0.683-1.314)	0.744			0.806(0.590-1.101)	0.176		
Obstructive jaundice Yes vs. no	0.923(0.533-1.600)	0.776			0.928(0.575-1.499)	0.761		
HBV infection Yes vs. no	0.722(0.515-1.012)	0.059			0.861(0.629-1.179)	0.351		
AFP (ng/ml) ≥20 vs. <20	0.856(0.549-1.333)	0.491			1.051(0.702-1.575)	0.808		
CEA (ng/ml) ≥5 vs. <5	1.958(1.427-2.685)	<0.001			1.417(1.044-1.923)	0.025		
CA19-9 (U/ml) ≥37 vs. <37	2.067(1.464-2.920)	<0.001	1.520(1.037-2.228)	0.032	1.779(1.297-2.440)	<0.001	1.764(1.273-2.445)	0.001
ALT (ng/ml)	1.001(0.999-1.003)	0.292			1.001(0.999-1.002)	0.404		
AST (ng/ml)	1.003(1.000-1.005)	0.032			1.002(0.999-1.004)	0.127		
Albumin(ng/ml)	0.959(0.933-0.986)	0.003			0.966(0.941-0.992)	0.010		
Bilirubin(ng/ml)	1.003(1.001-1.005)	0.055			1.004(1.001-1.006)	0.002		
PT(s)	1.057(0.948-1.180)	0.318			1.028(0.927-1.139)	0.606		
INR	0.938(0.821-1.071)	0.342			0.959(0.873-1.054)	0.385		
APTT(s)	0.997(0.973-1.021)	0.782			0.991(0.970-1.013)	0.442		
Child–Pugh Grade Grade A vs. Grade B	2.146(1.216-3.789)	0.008						
SII High group vs. low group	1.574(1.126-2.201)	0.008			1.590(1.181-2.140)	0.002		
ALBI High group vs. low group	1.692(1.220-2.346)	0.002			1.980(1.291-3.038)	0.002		
SII+ALBI Grade Grade B vs.Grade A Grade C vs.Grade A	1.519(1.013-2.278) 2.717(1.701-4.341)	0.037 <0.001	1.347(1.013-2.053) 2.230((1.371-3.628)	0.037 0.001	1.493(1.091-2.042) 3.078(1.822-5.198)	0.012 <0.001	1.225(1.004-1.696) 2.355(1.359-4.082)	0.032 0.002
Tumor number 1 vs. >1	1.426(0.978-2.079)	0.065			1.853(1.306-2.628)	0.001	1.614(1.114-2.339)	0.011
Tumor size (cm) >5.0 vs. ≤5.0	1.402(1.013-1.939)	0.041			1.293(0.954-1.751)	0.097		
Tumor differentiation Moderate vs. well Poor vs. well	2.193(1.142-4.209) 3.258(1.578-6.729)	0.018 0.001	1.685(1.105-3.314) 2.654(1.244-5.662)	0.028 0.012	1.646(1.063-2.812) 2.333(1.251-4.350)	0.029 0.008	1.334(1.073-2.302) 2.068(1.089-3.925)	0.035 0.026
Perineural invasion Yes vs. no	1.691(1.232-2.322)	0.001			1.246(0.927-1.676)	0.145		
Microvascular invasion Yes vs. no	1.993(1.451-2.737)	<0.001	1.548(1.112-2.156)	0.010	1.473(1.092-1.987)	0.011	1.364(1.004-1.853)	0.047
AJCC 8th edition T stage T_2_ vs. T_1a_/T_1b_T_3_/T_4_ vs. T_1a_/T_1b_	1.342(0.947-1.903)	0.098			1.062(0.689-1.637)	0.784		
	1.514(0.982-2.332)	0.060			1.527(0.985-2.367)	0.058		
AJCC 8th edition N stage N_1_ vs. N_0_	1.840(1.307-2.592)	<0.001	1.452(1.011-2.085)	0.043	1.421(1.027-1.965)	0.034		
AJCC 8th edition M stage M_1_ vs. M_0_	1.620(0.400-6.556)	0.499			0.988(0.244-3.991)	0.986		

**Figure 3:**
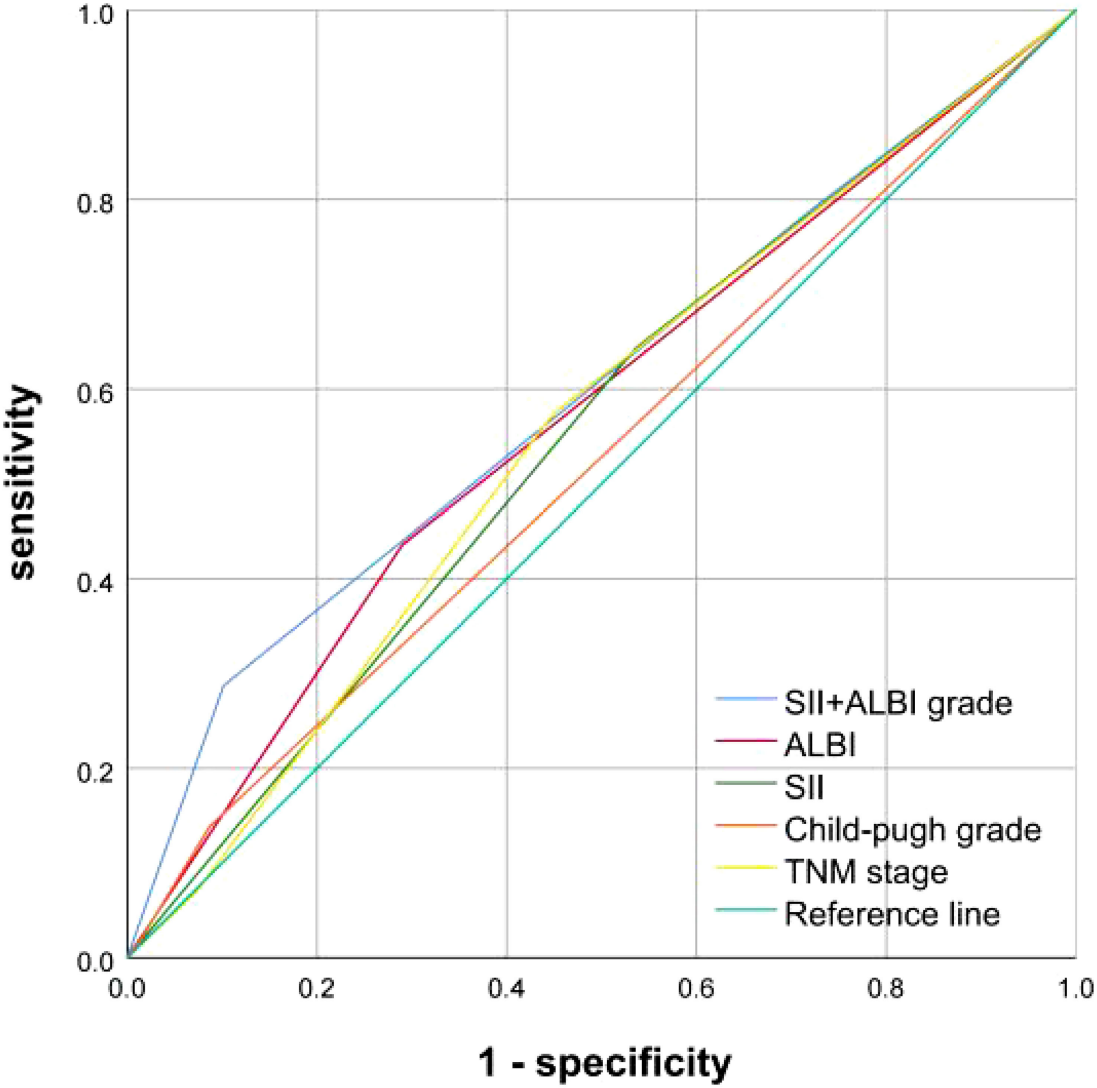
Comparison of SII, ALBI, SII+ALBI grade, Child-pugh Grade and TNM stage in predicting OS.

In addition, we plotted the ROC survival curves for SII, ALBI, SII+ALBI grade, Child-pugh Grade and AJCC 8^th^ TNM stage. By comparing the area under the ROC curves, we found that SII+ALBI grade demonstrated a superior survival predictive effect (**Figure 3**).

### 3.2 Correlation analysis of SII, ALBI and SII+ALBI with clinical and pathological features

Through chi-square tests, we found that compared to SII and ALBI, SII+ALBI grade exhibited better correlations with age, obstructive jaundice, HBV infection, CA19-9, CEA, Child–Pugh Grade, tumor size, tumor differentiation, perineural invasion (p<0.05, **Table 3**).

**Table 3:** Relationship of SII, ALBI and SII+ALBI grade with clinicopathological characteristics of intrahepatic cholangiocarcinoma (ICC) after radical resection in the training set.

	SII		X^2^	p-value	ALBI		X^2^	p-value	SII+ALBI Grade			X^2^	p value
	Low group	High group			Low group	High group			Grade A	Grade B	Grade C		
Sex			1.768	0.184			0.012	0.912				3.294	0.193
Male	60	80			97	43			44	69	27		
Female	41	77			81	37			26	70	22		
Age			0.911	0.340			0.002	0.964				7.010	0.030
≤65	61	104			114	51			38	99	28		
>65	40	53			64	29			32	40	21		
Obstructive jaundice			0.017	0.897			18.259	<0.001				10.157	0.006
No	83	130			159	54			62	118	33		
Yes	18	27			19	26			8	21	16		
HBV infection			6.834	0.009			3.805	0.051				11.015	0.004
No	56	112			109	59			35	95	38		
Yes	45	45			69	21			35	44	11		
AFP (ng/ml)			0.947	0.331			1.351	0.245				2.280	0.320
<20	83	136			148	71			56	119	44		
≥20	18	21			30	9			14	20	5		
CEA (ng/ml)			9.779	0.002			0.269	0.604				7.978	0.019
<5	76	88			115	49			54	83	27		
≥5	25	69			63	31			16	56	22		
CA19-9 (U/ml)			5.880	0.015			7.205	0.007				16.720	<0.001
<37	48	51			78	21			41	44	14		
≥37	53	106			100	59			29	95	35		
SII							0.008	0.930				156.891	<0.001
Low group					70	31			70	31	0		
High group					108	49			0	108	49		
ALBI			0.008	0.930								145.410	<0.001
Low group	70	108							70	108	0		
High group	31	49							0	31	49		
SII+ALBI grade			156.891	<0.001			145.410	<0.001					
Grade A	70	0			70	0							
Grade B	31	108			108	31							
Grade C	0	49			0	49							
Child–Pugh grade			0.171	0.679			54.684	<0.001				31.612	<0.001
Grade A	91	142			177	56			69	130	34		
Grade B	10	15			1	24			1	9	15		
Tumor Number			2.840	0.092			0.004	0.947				2.383	0.304
=1	87	122			144	65			61	109	39		
>1	14	35			34	15			9	30	10		
Tumor Size(cm)			19.244	<0.001			2.223	0136				7.382	0.025
≤5	58	47			67	38			38	49	18		
>5	43	110			111	42			32	90	31		
Tumor differentiation			1.062	0.588			5.498	0.064				6.736	0.151
Well	13	14			23	4			10	17	11		
Moderate	73	120			133	60			49	108	36		
Poor	15	23			22	16			11	14	2		
Perineural invasion			9.145	0.002			10.043	0.002				19.220	<0.001
No	67	74			109	32			52	72	17		
Yes	34	83			69	48			18	67	32		
Microvascular invasion			0.500	0.480			1.296	0.255				1.679	0.432
No	58	84			102	40			42	76	24		
Yes	42	73			75	40			27	63	25		
AJCC 8th edition T stage			7.729	0.021			3.851	0.146				3.737	0.443
T1a/T1b	55	62			80	37			38	59	20		
T2	35	60			61	34			22	52	21		
T3/T4	11	35			37	9			10	28	8		
AJCC 8th edition N stage			2.403	0.121			0.671	0.413				0.398	0.819
N_0_	79	109			127	61			53	100	35		
N_1_	22	48			51	19			17	39	14		
AJCC 8th edition M stage			0.043	0.836			1.804	0.179				1.244	0.537
M_0_	100	155			177	78			70	137	48		
M_1_	1	2			1	2			0	2	1		

### 3.3 Development and assessment of nomogram

**Figure 4:**
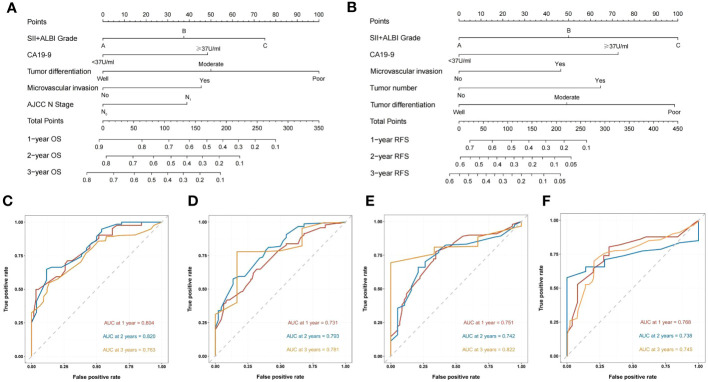
Construction and validation of the nomograms. Nomograms incorporating SII + ALBI Grade and other clinicopathological parameters for OS **(A)** and RFS **(B)** prediction in the training cohort. ROC survival curves of the training set for OS **(C)** and RFS **(D)** based on the model. ROC survival curves of the validation set for OS **(E)** and RFS **(F)** based on the model.

**Figure 5:**
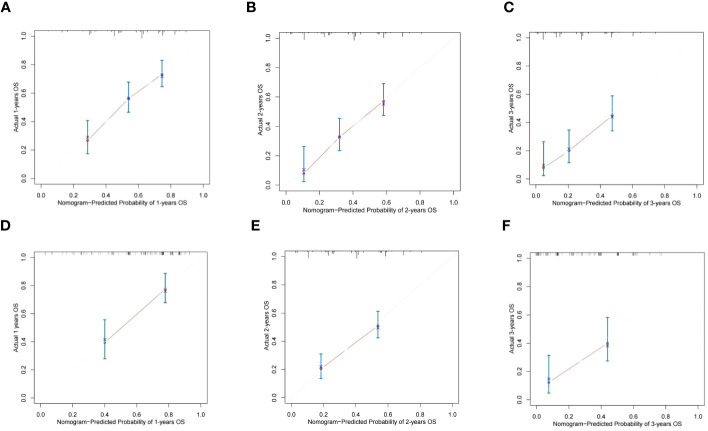
The calibration curves of the nomograms between predicted and observed 1-, 2-, and 3-year OS of patients in the training set **(A–C)** and the validation set **(D–F)**. The dashed line of 45° represents the perfect prediction of the nomogram.

**Figure 6:**
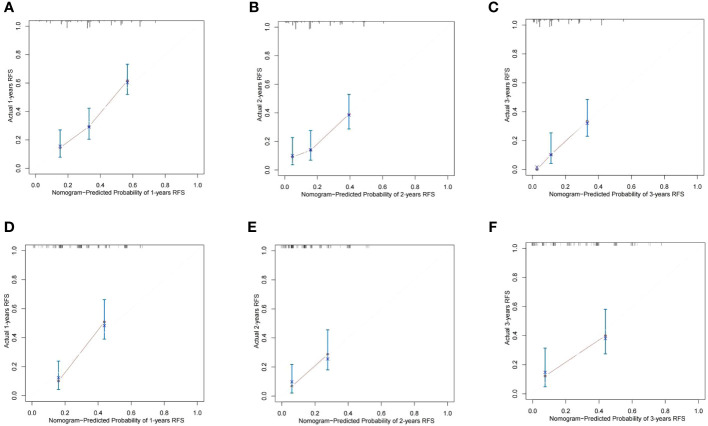
The calibration curves of the nomograms between predicted and observed 1-, 2-, and 3-year RFS in the training set **(A–C)** and the validation set **(D–F)**. The dashed line of 45° represents the perfect prediction of the nomogram.

Based on the results of Cox multivariate survival analysis, we established a nomogram prediction model using R software for postoperative OS and RFS in patients with ICC, incorporating various variables including SII+ALBI grade (**Figure 4**). In addition, we plotted the ROC survival curves for the training and validation sets based on the predictive model. The AUC values for 1–3-year OS in the training set were 0.804, 0.820, and 0.763, respectively, while for the validation set, they were 0.731, 0.793, and 0.781. The AUC values for 1–3-year RFS in the training set were 0.751, 0.742, and 0.822, respectively, and for the validation set they were 0.768, 0.738, and 0.745 (**Figure 4**). We also plotted the calibration curves of the training and validation sets for 1–3-year survival using both models, and the results consistently demonstrated the excellent predictive ability of the model for postoperative survival in ICC patients (**Figures 5**, **6**).

## 4 Discussion

Curative surgical resection represents the gold standard for the treatment of ICC (14). The decision to proceed with surgical resection is often based on the patient’s imaging data and the presence of accompanying symptoms. However, even among patients with similar disease stages and grades, there exists significant heterogeneity in the prognosis and clinical response to curative surgical resection (15). Therefore, the identification of a robust intraoperative and postoperative risk prediction tool holds paramount importance.

As a composite index of platelet, lymphocyte, and monocyte counts, SII provides a direct reflection of the body’s inflammatory status. Increasing evidence suggests that platelets and monocytes can interact with tumor cells through various mechanisms, promoting tumor cell survival and metastasis, enhancing cancer cell invasion, proliferation, and immune evasion, thereby modulating the interplay between the host and tumor (16–19). On the other hand, lymphocytes play a crucial role in cell-mediated immune destruction of cancer cells by activated T cells and other lymphocytes, while tumors can also release cytokines such as IFN-γ and TNF-α to regulate various immune functions in the body (20, 21). Furthermore, numerous studies have confirmed that SII is an independent prognostic factor for postoperative survival in various digestive system malignancies, including HCC, ICC, and gallbladder cancer (8, 22–25). Similarly, in our study, a lower SII was significantly associated with improved postoperative survival and reduced recurrence rates, further validating this observation.

Albumin-bilirubin, calculated based on serum albumin and bilirubin levels, provides an intuitive reflection of a patient’s immune status and liver function; ALBI was initially proposed by Johnson et al. in 2014 as an alternative to the Child-Pugh classification for assessing liver function in HCC patients, overcoming its limitations (26). Increasing evidence suggests that ALBI is a reliable indicator of liver functional reserve. A multicenter cohort study demonstrated that the predictive performance of the Barcelona Clinic Liver Cancer (BCLC) staging system based on ALBI score is comparable to or even superior to that based on the Child-Pugh classification (27). Subsequently, the predictive ability of ALBI for the prognosis of HCC and ICC patients has been validated in multiple independent cohorts, including those from Japan, China, and other countries (28–30). Consistent with the findings of these studies, in our research, the low ALBI group exhibited significantly higher OS and RFS rates compared to the high ALBI group.

In our study, we took into consideration the patients’ inflammatory status, immune capacity, and liver function, by combining SII and ALBI, which were categorized into three grades: A, B, and C. Through the construction of Kaplan-Meier survival curves and ROC survival curves, we found that the SII+ALBI grade had better predictive ability and discrimination when compared separately to SII and ALBI. Therefore, we included the SII+ALBI grade as an independent grade index in our model and confirmed that the nomogram predictive model incorporating SII+ALBI grade for OS and RFS demonstrated good predictive performance. Additionally, we analyzed the correlation between SII+ALBI grade and clinical and pathological characteristics. Surprisingly, for indicators such as microvascular invasion and 8th edition AJCC N stage, which showed no significant correlation with individual SII or ALBI, the SII+ALBI classification still exhibited a correlation. Therefore, we believe that the SII+ALBI classification can better reflect the patients’ clinical and pathological characteristics to a certain extent.

After reviewing relevant research, we found that our study is the first to combine SII and ALBI and construct a prognostic survival model based on SII+ALBI grade. In our model, SII+ALBI grade carries a significant weight, which is closely related to representing the immune-inflammatory status and liver function. Additionally, we plotted ROC survival curves and calibration curves for the training and validation sets based on the predictive model. The results demonstrated excellent predictive ability of the model for postoperative survival in patients with ICC.

In addition, our study has the following limitations. Firstly, although it is a multicenter retrospective study, the sample size involved in the study is relatively small, with a total of 374 cases. Secondly, due to the retrospective nature of this study, selection bias is unavoidable, and we only included patients who underwent surgical resection without receiving other treatments prior to surgery. Thirdly, despite our efforts to minimize the impact of confounding factors on the study results, individual differences in various laboratory parameters cannot be completely eliminated. Therefore, further large-scale prospective multicenter studies are still needed to validate our findings.

## 5 Conclusion

In conclusion, this multicenter study included a sample of 374 patients with ICC who underwent surgical resection in three tertiary hospitals. Based on univariate, multivariate, and clinical significance analyses, multiple relevant indicators incorporating the SII+ALBI grade were incorporated to construct a nomogram predictive model for OS and RFS. The model demonstrated excellent accuracy in survival prediction. To our knowledge, this is the first clinical prediction model for ICC that includes the SII+ALBI grade. We believe that this model can provide better guidance for the management of ICC and has the potential for broad application.

## Data availability statement

The raw data supporting the conclusions of this article will be made available by the authors, without undue reservation.

## Ethics statement

This study received ethical approval from the Institutional Review Boards of Zhengzhou University People’s Hospital (Ref No. 2023-012), Zhengzhou University Cancer Hospital (Ref No. 2023-203), and Zhengzhou University First Affiliated Hospital (2021-KY-1137-002). Written informed consent was obtained from all patients prior to their participation in the study. The studies were conducted in accordance with the local legislation and institutional requirements.

## Author contributions

HZ is the first author. HZ, QL and HY conceived and designed the study. HZ, GH, ZY, KC and BM collected and offered the data. HZ, GH and ZY performed follow-up and statistical analysis. HZ wrote the manuscript. HY critically revised the manuscript for important intellectual content. All authors contributed to the article and approved the submitted version.
